# Consensus conference on platelet transfusion: final statement.

**DOI:** 10.1038/bjc.1998.488

**Published:** 1998-08

**Authors:** 


British Journal of Cancer (1998) 78(3). 290–291
© 1998 Cancer Research Campaign

### Consensus conference on platelet transfusion

### Final Statement

Royal College of Physicians of Edinburgh. 9 Queen Street. Edinburgh EH2 IJQ. UK

### INTRODUCTION

Platelet transfusion is used in patients with a low platelet count (thrombocytopenia) or disordered platelet function who are actively bleeding (therapeutic use) or who are at serious risk of bleeding (prophylactic use). There is extensive clinical evidence that platelet transfusions are valuable but the procedure also carries risks and costs. The use of platelet transfusion has developed without much clinical trial-based evidence on its effects and complications or on the value of additional procedures. such as leucocyte depletion. This has obvious implications for the ethically crucial questions of patient safety and the balance of risks and costs to benefits. Despite the difficulties in performing randomized clinical trials on an established procedure. the conference is firmly of the view that such studies are needed to allow clearer. evidence-based guidelines for the use of platelet transfusion to be formulated. Even when randomized trials are not possible. the conference recommends that all decisions to transfuse platelets should be made according to written institutional protocols that will allow generally applicable conclusions to be drawn from the results.

### INDICATIONS FOR PLATELET TRANSFUSION

### Prophylactic

The main use of platelet transfusion is in the prevention of bleeding in patients with haematological malignancies (particularly leukaemias) who have bone marrow failure caused by their disease or its treatment.

For patients with bone marrow failure. it has been accepted practice to transfuse platelets when levels are very low. Based on the evidence presented at the meeting. there was a general agreement that a platelet threshold of $10 \times 10^9$ per litre is as safe as higher levels for treating most patients without additional risk factors. These risk factors. which include sepsis. concurrent use of drugs (e.g. antibiotics) and other abnormalities of haemostasis. are indications for a higher threshold. Higher threshold numbers are also needed to cover invasive procedures. e.g. line insertions and biopsies. but there is no consensus on appropriate thresholds. For uncomplicated patients. evidence on the safety of even lower platelet thresholds than $10 \times 10^9$ per litre should be sought. However. accurate counting of low platelet numbers may create difficulties when trying to reduce the threshold below $10 \times 10^9$ per litre. In neonates. in whom there is a considerable danger of haemorrhage. platelet transfusion is indicated at a higher threshold than in adults.

Received 7 January 1998
Accepted 7 January 1998

Correspondence to: M Contreras. Royal College of Physicians of Edinburgh

The avoidance of low haematocrit in patients with thrombocytopenia or disordered platelet function reduces the risk of haemorrhage.

### Therapeutic

Most major surgery (including cardiac and vascular) can be successfully carried out without platelet transfusions. Patients who have taken aspirin in the 10 days before surgery have increased risk of bleeding and the value of continuing aspirin at this time should be evaluated.

In massive haemorrhage. the first priorities are to achieve surgical haemostasis and resuscitation. There is a consensus to transfuse platelets if the count is less than $50 \times 10^9$ per litre. but it is clear that other clinical criteria need to be considered.

Liver transplantation gives special problems with haemostasis. and platelet transfusion is frequently required. In liver surgery. thromboelastography has been shown to be a good predictor of platelet need and deserves evaluation in other areas. Antifibrinolytic agents have a platelet-sparing role in liver transplantation and complex cardiac surgical operations. They may also be used in other situations. as may other agents. such as DDAVP. when platelet numbers or function are compromised.

Platelet transfusion is also used to treat clinically significant bleeding in patients with idiopathic thrombocytopenic purpura (ITP). The consensus view is that platelet transfusions have been over-used in this area. especially in children. Intracranial or eye haemorrhage or severe bleeding from the gut are the major concerns. Except in these circumstances. platelet transfusion should be avoided.

### RISKS AND CONTRAINDICATIONS

The most common acute risk is bacterial infection. which has been under-reported. The important long-term risks of transmitting viral infection from the donor are well recognized and have been substantially reduced. However. the threat from newly recognized viruses is always present. In this connection the theoretical possibility that blood products may be able to transmit the agent of variant Creutzfeldt–Jakob disease (CJD) has attracted attention and cannot be discounted. Experimental procedures for reducing the risk of transmitting bacterial and viral infection by chemical treatment of the platelet preparations (e.g. by Psoralens plus ultraviolet light) are under investigation.

The presence of white blood cells in the platelet preparation enhances the risk of certain viral infections and is largely responsible for generating cytokines. which can give rise to febrile reactions after platelet transfusion. Platelet transfusion through negatively charged filters can also activate the contact system and give rise to significant hypotension in patients receiving angiotensin-convesting enzyme (ACE) inhibitors. A tenfold reduction in white cell numbers (leucoreduction) before storage of platelets is sufficient to abolish most febrile reactions and is readily achievable in both buffy coat-derived and apheresis platelets. White cell numbers can be further reduced to less than $5 \times 10^6$ per dose (leucodepletion) by specialized manufacture, by improving the apheresis technique and by pre-storage filtration. Leucodepleted platelets reduce transmission of some viruses and febrile reactions, as well as reducing HLA allo-immunization, which can cause patients to become refractory (resistant) to further platelet transfusions.

Irradiation of platelet concentrates is required to prevent graft vs host disease, and this needs to follow national guidelines.

Platelet transfusion is contraindicated in heparin-induced thrombocytopenia, thrombotic thrombocytopenic purpura and the haemolytic uraemic syndrome.

### RECIPIENTS WHO ARE REFRACTORY TO PLATELET TRANSFUSIONS

Failure to achieve a satisfactory response to platelet transfusion (refractoriness) occurs in up to half of those receiving prophylactic transfusions. This is defined by the poor increment in platelet count rather than on clinical grounds. Patients who remain refractory for immunological reasons, often receive prophylactic transfusions with HLA-matched or crossmatch-compatible platelets. Satisfactory increments are frequently obtained by these approaches, but their effectiveness in reducing severe bleeding deserves more detailed evaluation. This also applies to the relative effectiveness of HLA matching compared with platelet cross-matching, and whether patients who remain refractory despite these measures should only be transfused therapeutically.

### QUALITY CONTROL OF PLATELET PREPARATIONS

Further quality control measures should become routine in addition to those already undertaken and should include more accurate measurements of low white cells and high platelet counts, microbiological safety as well as rapid techniques for detecting viral nucleic acid in platelet preparations. There are few appropriate surrogate markers that are relevant to the arrest or prevention of bleeding in patients, although the swirl test has its adherents.

Performance in quality assurance should meet evolving national standards.

### CONCLUSION

The overall conclusion is that platelet transfusion is a well-established clinical procedure, but it can never be entirely safe and must be given only when there is clear clinical justification. Nevertheless, the precise indications for its use and the optimal specification of the product still need to be defined. These goals will be achieved only by a combination of prospective randomized trials with the information that will come from the use of clearly defined protocols and their efficient audit.

© Cancer Research Campaign 1998


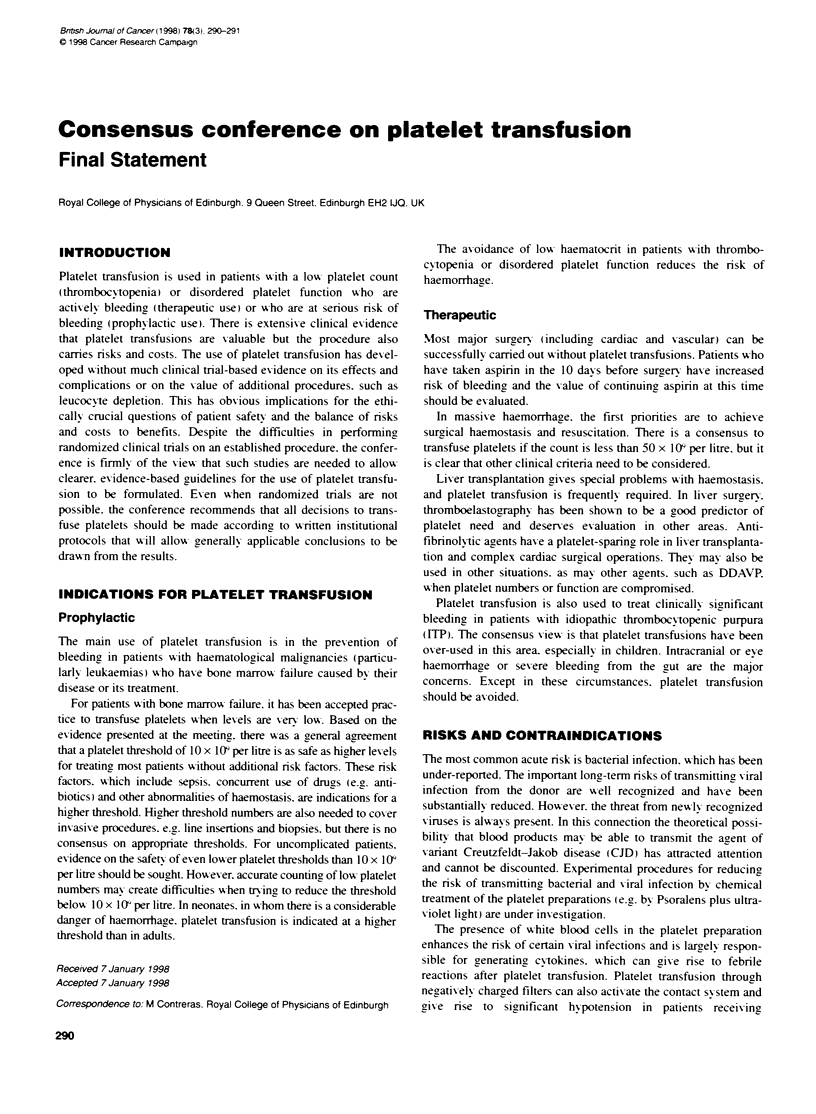

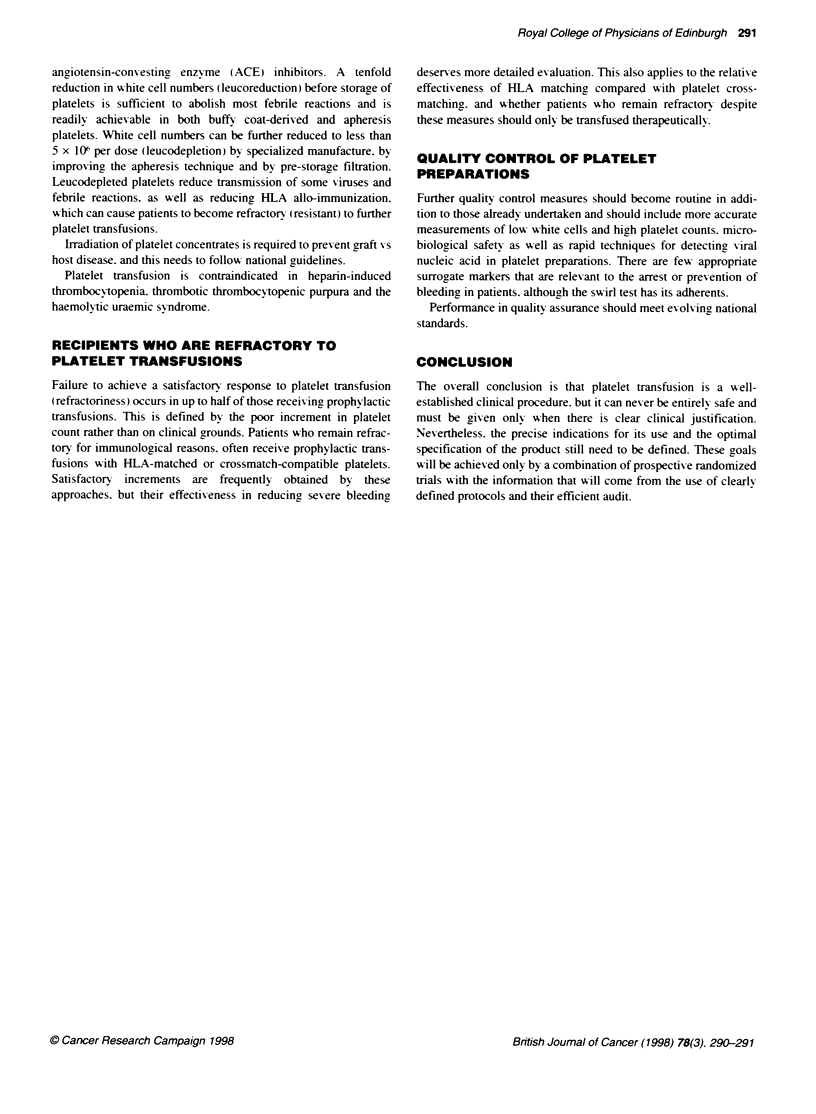


## References

[OCR_00050] Hayhurst E. R., Albaugh R. P., Holmes P. M., Starr E. B. (1920). INDUSTRIAL HYGIENE AND OCCUPATIONAL DISEASE.. Am J Public Health (N Y).

